# Significance of scattered small echogenic foci floating in urinary bladder as ultrasonography finding in dogs

**DOI:** 10.1186/s12917-024-04008-9

**Published:** 2024-08-08

**Authors:** Hamidreza Moosavian, Majid Masoudifard, Maede Beiki Zareh, Shahram Jamshidi, Iraj Ashrafi Tamai

**Affiliations:** 1 Department of Clinical Pathology, Faculty of Veterinary Medicine, University of Tehran, Tehran, Iran; 2 Department of Surgery and Radiology, Faculty of Veterinary Medicine, University of Tehran, Tehran, Iran; 3 Department of Internal Medicine, Faculty of Veterinary Medicine, University of Tehran, Tehran, Iran; 4 Department of Microbiology and Immunology, Faculty of Veterinary Medicine, University of Tehran, Tehran, Iran

**Keywords:** Echogenic foci, Urinary bladder, Ultrasonography, Urinalysis, Urine culture, Dogs

## Abstract

**Background:**

Despite the prevalence of echogenic foci floating in the urinary bladder seen in ultrasonography in dogs, surprisingly little has been written on its significance, including its potential association with urinalysis. The objective of the study was to determine the diagnostic value of the echogenic foci floating in urinary bladders in dogs.

**Results:**

- Cystosonography was performed on 45 dogs. Bladder contents were examined and divided into positive (containing echogenic particles) and negative (absent echogenic particles) groups according to the presence and absence of floating echogenic particles. Five mL of urine was collected via cystocentesis. Urine analysis and culture were done and the relationship between ultrasound evaluation and urinalysis results was investigated. In dogs with bladder echogenic particles in ultrasonography, the prevalence of hematuria, pyuria, bacteriuria, and lipiduria were 88.9%, 92.6%, 29.6%, and 70.3%, respectively. However, in dogs in which echogenic particles were not observed in their bladders, the prevalence of hematuria, pyuria, bacteriuria, and lipiduria was 77%, 50%, 5.5%, and 77%, respectively. There was a significant association between bladder debris and positive urine culture, with an odds ratio of 7.15 (95% confidence interval: 0.81–63.28) compared with matched controls. Furthermore, there was a significant relationship between the presence of floating echogenic particles with pyuria, and urine color ( *p* ≤ 0.05).

**Conclusion:**

In conclusion, the present results showed the detection of bladder debris on ultrasound can be a predictor for pyuria and positive urine culture in dogs.

## Background

In small animal medicine, various pathologic conditions can affect the urinary tract and cause clinical signs such as hematuria, pollakiuria, and stranguria [1]. However, in some patients, urinary tract disorders may show no remarkable clinical signs. On the other hand, none of the clinical signs is indicative of a specific disorder. Ultrasonography is a noninvasive and effective method for urinary tract assessment in small animals and provides good information in real time [2]. Various abnormalities in the urinary bladder such as urine calculi, cystitis, polyps, and cancer can be assessed by ultrasonography [3]. In ultrasonography, a normal fluid-filled urinary bladder is uniformly anechoic, with well-defined walls, and no echogenic foci are seen. In some patients, echogenic foci as floating or layering in a gravity-dependent manner may be seen within the lumen of the urinary bladder [4]. The abnormal debris can be caused by a technical artifact or a pathologic condition [5]. The detection of echogenic foci floating in the urinary bladder during ultrasonography can be indicative of various pathologic conditions such as hematuria, pyuria, crystalline matrix, and fat droplets [6]; however, the importance of these particles and their correlation with urinalysis findings in dogs are not widely discussed in the literature. The objectives of this study were to determine associations among scattered small echogenic foci floating in the urinary bladder in the ultrasonography and urinalysis findings in the dogs.

## Methods

Study design and patient selection

We performed a cross-sectional study of 45 dogs who presented to the Veterinary Teaching Hospital of the University of Tehran between 2022 and 2023 and underwent both urinary tract ultrasonography and urinalysis with urine culture on the same day. The dogs identified with bladder debris in ultrasonography were matched to controls based on breed, age, and gender. Age-matching of controls to subjects was conducted within a range of ± 1 years.

### Data collection

Dogs were included if they had urinary tract ultrasonography and cystocentesis on the same day and were excluded if they had recent antibiotic therapy, genitourinary surgery, or parturition. In all animals, hair was clipped, and urinary tract ultrasonography was performed in awake animals in dorsal recumbency using a 5–12 MHz linear probe (Philips Affiniti 70). For optimal contact, ultrasound gel was applied to the skin and probe. Effects made to use constant conditions of frequency, TGC (Time, gain, compensation), brightness, and contrast of the sonographic image. The presence or absence of echogenic foci floating in the bladder lumen and any other abnormal findings were recorded by an expert radiologist (DVM, DVSc). In each dog, a sterile urine sample was taken through cystocentesis and analyzed immediately (< 1 h) after collection. Urine specific gravity (USG) was measured by a refractometer. Urine chemical properties such as blood, pH, and glucose were analyzed by urine test strips, and finally, microscopic examination was performed on urine sediment. Briefly, 5 mL of well-mixed urine was centrifuged at 500 g for 5 min, and then 4.5 mL of supernatant was removed, and 0.5 mL of the supernatant (10% of the original aliquot volume) was used to resuspend the sediment pellet, to create a suspension. This suspension was then subjected to microscopic examination. The presence of casts and crystals was evaluated under low magnification, while the presence of leukocytes, erythrocytes, and fat droplets was assessed under high magnification using microscopy. A scoring system was employed to assess epithelial cells, leukocytes, erythrocytes, and fat droplets in urine. For leukocytes, the numbers were categorized as 0–3, 3–10, 10–15, 15–20, and more than 20, and scored from 0 to 4, respectively. For erythrocytes, and transitional cells, the numbers were categorized as 0–5, 5–10, 10–15, 15–20, and more than 20, and scored from 0 to 4, respectively. For fat droplets, the numbers were categorized as 0, 1–10, 10–20, 20–30, and more than 30, and scored from 0 to 4, respectively. The presence of casts and crystals was reported as either positive or negative.

### Bacterial isolation

A portion of the urine was aseptically sent for bacterial culture. For urine culture and bacterial isolation, 50 µl of urine specimens collected by cystocentesis were inoculated on sheep blood agar and MacConkey lactose agar plates at 37 °C for 24–48 h. For further evaluation, subcultures of the resulting growth made on blood agar, Gram’s reaction, morphology, and colony characteristics, catalase and oxidase tests were performed. Eosin methylene blue agar was used to differentiate Escherichia coli from other gram-negative pathogens.

### Statistical analysis

Statistical analysis was performed using PASW Statistics for Windows release 21 (SPSS Inc., Chicago, IL, USA). The chi-square test was used to evaluate the relationship between the presence of echogenic particles and urinary findings and urine culture results. Mann-Whitney and Student’s t-test were used to study the changes of each analyte between two groups of dogs with or without bladder debris. *p* < 0.05 was considered significant.

## Results

In the present study, the cases and controls were individually matched on age, sex, and breed. On average (Mean ± SE), control dogs and dogs with bladder echogenic particles were 38.27 ± 9.57 and 32.55 ± 5.96 months, and weighed 16.44 ± 1.55 and 15.90 ± 1.7 kg, respectively.

In the control group, there were 10 female dogs and 8 male dogs and in the group of dogs with echogenic urine, there were 13 female dogs and 14 male dogs. There was no significant correlation between gender and the presence of echogenic particles, as well as between urine culture results and gender.

**Fig. 1:**
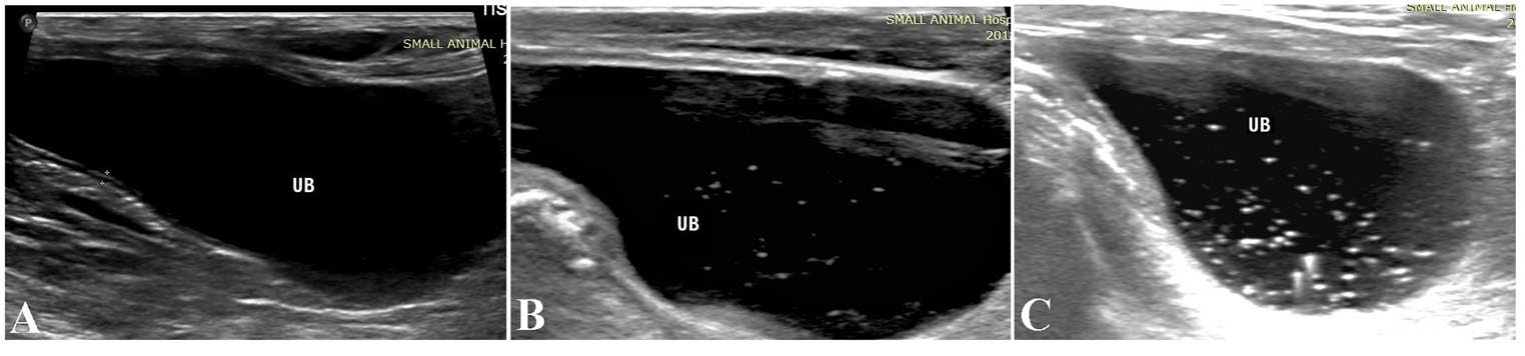
(**A**) Normal urinary bladder with anechoic urine. (**B**) and (**C**) Suspended echogenic foci in moderate (**B**) and large (**C**) numbers are visible in each urinary bladder

In the ultrasound evaluation of the urinary bladder, out of 45 dogs, in 18 dogs (40%) the bladder contents were completely anechoic (Fig. 1A), and in 27 dogs (60%) floating hyperechoic foci were found (Fig. 1B, and C). Out of the 45 dogs studied, in 40 dogs (88.8%), the kidney was normal and in 5 cases (11.1%) abnormal findings (such as size change, presence of cysts, echogenicity changes in the medullary and cortical parts) were observed. The urinalysis results showed, 15 samples (33.3%) had a clear appearance, of which 6 (40%) had floating foci and 9 (60%) had no floating foci in ultrasound evaluation. 15 samples (33.3%) had a semi-transparent appearance, of which 10 (66%) were in the positive group and 5 (33%) were in the negative group. 15 samples were cloudy, of which 11 (73%) of them were in the positive group and 4 (26%) of them were in the negative group. In terms of pH, the urine samples were in the range of 5–9 and the USG of all urine samples were in the range of 1.010 to 1.055.

There was no significant correlation between the echogenicity of urine in sonography and urine pH results. The pH (Mean ± SE) in the non-echogenic urine group was 6.16 ± 0.25, while in the echogenic group, it was slightly higher at 6.5 ± 0.15.

**Fig. 2:**
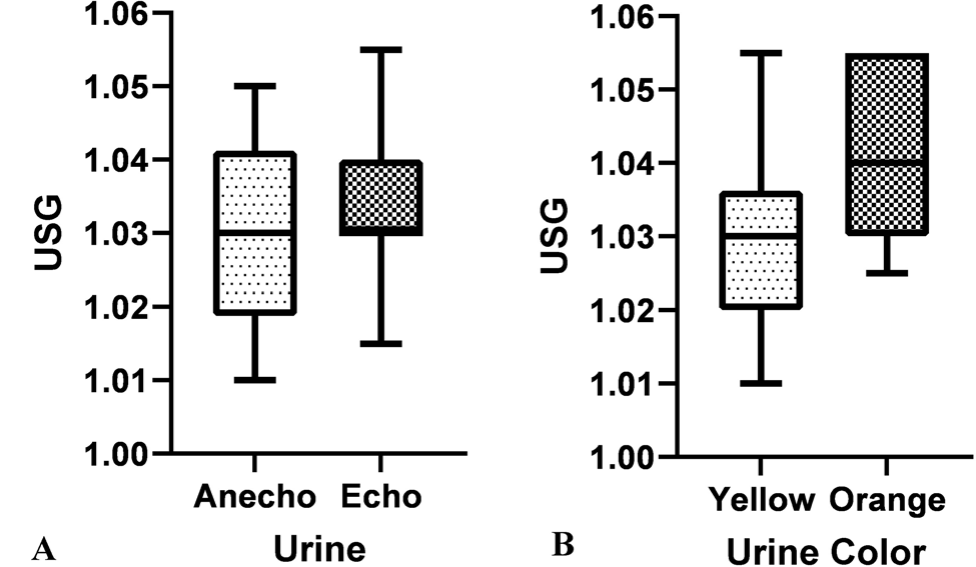
Changes in urine specific gravity based on urine echogenicity on sonography (**A**) and urine color on macroscopic evaluation (**B**). (**A**) There are no significant differences in USG in anechoic and echoic urine. (**B**) The orange urine had a higher USG than the yellow urine, significantly

There was no significant correlation between urine echogenicity in ultrasound and USG (Fig. 2A). The color of urine in 37 samples (82%) was yellow, of which 19 (51%) had echogenic foci and 18 (48%) had no echogenic foci in ultrasound evaluation. 7 urine samples (15%) were orange, all of which were in the positive group.

There was a significant difference in USG between yellow and orange colors as the urine with orange color had a higher USG than yellow color, significantly (Fig. 2B). One urine sample was reported as colorless and contained echogenic points in ultrasound. 36 of the samples were negative in terms of bacterial culture and 9 were positive in terms of bacterial culture. Among the 9 samples that had positive bacterial culture, 8 cases showed the presence of hyperechoic floating foci in their ultrasonography evaluation, and no hyperechoic floating foci were observed in only 1 sample (Table 1). According to the results obtained by the correlation test, the presence of hyperecho-floating foci in the bladder has a significant relationship with urine color and bacteriuria. There was a significant association between bladder debris and positive urine culture, with an odds ratio of 7.15 (95% confidence interval: 0.81–63.28) compared with matched controls. Furthermore there was a significant correlation (*p* < 0.05) between pyuria and microbial culture results. In all cases where positive microbial culture results were reported, pyuria was also positive., which reduces the likelihood of contamination.

**Table 1:** Urine culture results for dogs with and without bladder debris on renal bladder ultrasound (*p* < 0.05)

Urine culture		Bladder debris (*n* = 27)			No bladder debris (*n* = 18)	
	Positive	8 (29.6%)	*E. coli* (4) *S.P* (2) *P.V* (1) *T.P* (1)		1 (5.6%)	*E. coli* (1)
	Negative	19 (70.4%)			17 (94.4%)	

E. coli: Escherichia coli, S.P: Staphylococcus pseudintermedius, P.V: Proteus vulgaris,, T.P: Trueperella pyogenes

No significant relationship was observed between, urine turbidity, cast, and crystal with the presence of floating echogenic foci in the bladder.

Overall, crystalluria was observed in 9 dogs, which accounted for 20% of the total population. In the non-echogenic urine group, crystalluria was observed in 3 dogs (3/18, 16.6%), while in the echogenic urine group, it was observed in 6 dogs (6/27, 22.2%). In all cases, only struvite crystals were reported.

In dogs with bladder echogenic particles in ultrasonography, the prevalence of hematuria, pyuria, and lipiduria were 88.9%, 92.6%, and 70.3%, respectively. However, in dogs in which echogenic particles were not observed in their bladders, the prevalence of hematuria, pyuria, and lipiduria were 77%, 50%, and 77%, respectively. There was a significant relationship between the presence of floating echogenic particles with white blood cells (*P* ≤ 0.05).

## Discussion

Ultrasound of the urinary bladder as a safe, rapid, and inexpensive diagnostic imaging technique is the modality of choice for imaging the majority of disorders affecting the urinary bladder [7]. During abdominal ultrasound, echoes may be seen in the urinary bladder, attributed to hematuria, pyuria, crystalluria, and lipiduria. However, there were rare studies to show the correlation between the finding of echoes on ultrasound and the urinalysis results in dogs. In a recent study, 19% of dogs were reported as having echoic urine in ultrasound exams. In that study, suspended material with or without reverberation and gravity-dependent sediment with or without acoustic shadowing in the urinary bladder were defined as echoic urine [8].

Lipiduria in cats is one of the most important known causes that can appear as suspended echoes in the urinary bladder on ultrasonographic examination [6]. Lipiduria is a common physiological phenomenon in cats that can be detected on routine urinalysis as an incidental finding. Exfoliation of renal cortical proximal convoluted tubules accumulated with lipid droplets into the urine is thought to be the cause of lipiduria in cats [9]. Abundant lipid accumulation in renal cortices and lipiduria have been associated with hyperechoic feline renal cortex and suspended echoes in the urinary bladder on ultrasonographic examination, respectively [10]. In the present study, lipiduria was observed in the urinalysis of 70.3% of the patients with urinary bladder echoes and in 77% of patients without urinary bladder echoes during ultrasound exams. So, there was no significant correlation between lipiduria and urinary bladder echoes in the ultrasound exam. Studies in cats show this relationship can also be affected by the type of lipid. Increased amounts of urine diacylglycerol can have a positive effect on the presence of echoes seen on ultrasound [6].

In the urinalysis of 45 samples, pyuria was confirmed in 34 samples (75.5%), of which 25 cases had floating echogenic foci in ultrasound, and 9 cases had no echogenic foci. Out of 45 samples, 39 samples were negative for bacterial culture and positive for 9 cases. Out of 9 cases with positive bacterial cultures, echogenic foci were reported in bladder ultrasound in 8 cases. Statistically, there was a significant correlation between pyuria and bacteriuria in the urinalysis and the presence of echogenic foci in the ultrasonography. In a previous study, hyperechoic particles in the urinary bladder were observed in 12 cases out of 32 dogs diagnosed with urinary tract infection [11]. Sanchez et al. (2019), evaluated the relationship between urine echogenicity in ultrasound with urine sedimentation. In that study, urinary bladder ultrasonography was performed on 194 dogs and cats. The appearance of urine in ultrasound was divided into two categories including echoic and anechoic urine. Furthermore, cystocentesis and urinalysis were performed on the patients. Urine sediment was divided into two categories: active (presence of bacteria, blood, pus, and crystals) and inactive. In that study, 52 cases had echogenic urine and 142 cases had anechoic urine in ultrasound evaluation. Urine sedimentation was active in 52 cases and inactive in 142 cases. Also, according to the results of the study, anechoic urine had inactive sediment and was related to the absence of crystalluria, hematuria, and bacteriuria. The result of the study also showed a significant relationship between the presence of crystals in urinalysis and echogenicity of the bladder, but most of the cases that had active sediments showed bacteriuria in urine analysis [8]. Another study in cats with pyelonephritis showed echogenic debris within the renal pelvis is one of the important ultrasonographic findings [12].

In the present study, no significant correlation was found between the presence of echogenic foci on sonography and hematuria. In a study conducted by Kim et al. (2016) in humans, although the presence of hematuria was significantly higher in patients with echogenic urine, echogenic spots in the bladder were not observed in over 70% of patients with hematuria. The authors concluded that the presence of blood cells or clots may be a cause of the formation of echogenic foci in the bladder [13]. Probably, the intensity of hematuria, simultaneous presence of blood clots, or possibly concurrent inflammation are likely determining factors in urine echogenicity in patients with hematuria. Studies conducted in cats have shown that echogenic accumulations in the urinary bladder can be observed as a result of cellular debris and crystalline matrix, which are associated with conditions such as idiopathic feline cystitis [14]. However, in the present study, no correlation was found between urinary crystalluria and the formation of echogenic spots in the bladder. These results are consistent with some other findings that do not consider urinary crystalluria as a significant factor in urinary echogenicity [15, 16]. However, it appears that the small sample size in the current study and the lack of observation of various types of urinary crystals in patients have prevented definitive conclusions from being drawn on this matter. In the present study, only struvite crystals were observed in all cases of urinary crystalluria.

Interestingly, there was a significant correlation between urine color and urine echogenicity in the population. In the current data set, urine samples with orange color were always echoic. The relationship between urine color and urine echogenicity is likely due to the USG. Although USG does not directly correlate with urine echogenicity, all orange urine samples had a high USG and were bacteria-free.

A total of 9 dogs were identified which had bacteriuria, and Escherichia coli (5/9 (56%) was the most frequent organism, while Staphylococcus pseudointermedius 2/9 (22%), Proteus vulgaris 1/9 (11%), and Trueperella pyogenes 1/9 (11%) populated the remaining cultures.

Similar to the findings of the previous studies, the present study showed the most common cause of urinary tract infection in dogs, is uropathogenic Escherichia coli [17–19].

In a study conducted by Ourych et al. in 2022 on 1233 urine samples, E. coli, Enterococcus, Staphylococcus, and Proteus were identified as the most common bacterial species, respectively [20]. . In the present study Trueperella pyogenes has been isolated from a dog. Trueperella pyogenes (formerly called Arcanobacterium pyogenes), is a commensal bacteria of the mucous membranes of domestic animals, such as cattle, sheep, swine, and goats. However, it can also act as an opportunistic pathogen and is responsible for causing various types of pus-forming infections, including metritis, mastitis, pneumonia, and abscesses, which result in substantial economic losses in livestock breeding [21]. Rarely, this bacterium has been reported as a potential cause of urinary tract infections in dogs [22]. In the present study, it was isolated from a dog with urinary tract inflammation.

The results showed a significant relationship between the presence of urine-positive culture and urine echogenicity.

Out of 9 positive samples based on bacterial culture, echogenic foci was observed in the 8 samples in the urinary bladder ultrasonography. Furthermore, in all cases of positive bacterial culture, the presence of pyuria was simultaneously confirmed. A significant correlation was also observed between pyuria and the presence of echogenic particles, as well as between pyuria and positive microbial culture. The presence of aseptic conditions during sample collection and transfer, the concurrent presence of pyuria in urine analysis, and the isolation of a large number of pure colonies in positive samples reduce the likelihood of secondary contamination in the current study.

The present study had several limitations. First, the number of dogs studied in each group was relatively small. Increasing the number of dogs studied including both normal dogs and dogs with various urinary tract diseases would have resulted in a better understanding of the relationships between sonographic findings and urine analysis. Contrary to initial expectations, there was no correlation between urine echogenicity on sonography and urinary crystalluria. The lack of correlation between urinary crystals and the presence of echogenic foci in the present study may be due to the relatively small sample size. Evaluating a greater number of positive samples in terms of echogenic foci in bladder ultrasonography, as well as assessing dogs with different types of urinary crystals, can provide valuable information in understanding the nature of echogenic spots in the bladder. Additionally, since the appearance of these spots in ultrasonography can vary, in future studies, the correlation between their visual morphology and laboratory findings can be investigated. Second, due to the lack of a gold standard test for definitive diagnosis of the nature of echogenic foci in the bladder, the clinical implication of the positive echogenic sign observed could not be certainly suggested. Furthermore, it is also not possible to determine the sensitivity and specificity of sonographic findings. Third, the intensity of echogenic foci on sonography has not been quantitatively scored. Assessing the severity of the ultrasonography abnormality and follow-up urine analysis and ultrasonography after treatment could play key roles in determining the nature and the clinical implication of the urine echogenic debris in each subject. Despite these limitations, our study adds valuable information about the correlation of urine echogenic debris and urinalaysis findings. It highlights in dogs with floating hyperechoic foci in urinary bladders, there is a higher likelihood of the presence of pyuria and infection.

## Conclusion

Although ultrasonography is a valuable diagnostic tool for urine and urinary bladder evaluation, urinalysis is still necessary to characterize echogenic foci in the urinary bladder. In our study on dogs, a significant relationship was observed between pyuria and bacteriuria present in urinalysis and echoes observed in bladder content ultrasonography. We conclude that particulate echoes within the bladder during sonography may indicate the presence of urinary tract infections in dogs. However, the low number of dogs in the present study was an influential limitation. To evaluate other factors, a broader study on a larger number of dogs is needed.

## Data Availability

The data used and/or analyzed during the current study are available from the corresponding author on reasonable request.

## Abbreviations

MHz: Megahertz
TGC: Time, gain, compensation
USG: Urine specific gravity
SPSS: Statistical package for social sciences
SE: Standard error

## Declarations

All methods are reported in accordance with Arrive guidelines (https://ariveguidelines.org) for the reporting of animal experiments. All phases of this study were approved by the Animal Ethical Guidelines of the Faculty of Veterinary Medicine, University of Tehran, Tehran, Iran (No: 8509019.7.39).
